# Preferences for advisor agreement and accuracy

**DOI:** 10.1371/journal.pone.0311211

**Published:** 2024-09-27

**Authors:** Matt Jaquiery, Nick Yeung

**Affiliations:** Department of Experimental Psychology, University of Oxford, Oxford, United Kingdom; Fundación Universitaria del Área Andina, COLOMBIA

## Abstract

Previous research has shown that people are more influenced by advisors who are objectively more accurate, but also by advisors who tend to agree with their own initial opinions. The present experiments extend these ideas to consider people’s choices of who they receive advice from—the process of source selection. Across a series of nine experiments, participants were first exposed to advisors who differed in objective accuracy, the likelihood of agreeing with the participants’ judgments, or both, and then were given choice over who would advise them across a series of decisions. Participants saw these advisors in the context of perceptual decision and general knowledge tasks, sometimes with feedback provided and sometimes without. We found evidence that people can discern accurate from inaccurate advice even in the absence of feedback, but that without feedback they are biased to select advisors who tend to agree with them. When choosing between advisors who are accurate vs. likely to agree with them, participants overwhelmingly choose accurate advisors when feedback is available, but show wide individual differences in preference when feedback is absent. These findings extend previous studies of advice influence to characterise patterns of advisor choice, with implications for how people select information sources and learn accordingly.

## Introduction

The world is full of opinions and suggestions and recommendations, from a colleague’s helpful pointers on a paragraph to an advertisement’s insistence that you would look sexier in a more expensive pair of jeans. Because advice is ubiquitous, choosing where to obtain it is a decision with meaningful consequences: advice is optimally useful where it differs from the ideas we already have while still being based on objective and inter-subjective realities [1]. Continually seeking only advice that confirms already-held opinions has been argued to lead to the formation of echo chambers and drive political polarisation [2, 3].

The present research investigates this process of choosing advice, building conceptually and methodologically on the large body of existing research that has focused on the process of using advice—i.e., on identifying the factors affecting whether advice, once offered, influences a decision maker [4]. Studies of advice utilisation have shown that decision makers tend to downweight advice relative to their own opinions (termed “egocentric discounting” [5]), a tendency that is amplified when they are confident in those opinions [6–10] and reduced when the advice itself is expressed confidently [1, 11–13]. Advice utilisation also critically depends on decision makers’ degree of trust in their advisors: Advice from experts is more influential than from novices [1, 5, 11, 14–17], and people rapidly form impressions of advisors—both positive and negative—based on the experienced quality of the advice they provide [5].

A previous line of work from our lab indicated that people tend to develop greater trust in, and therefore be more influenced by, advisors who agree with them more frequently [18]. This effect is particularly strong when objective feedback is absent, but is still apparent when feedback is available (and hence when advice received can be evaluated against an objective standard). Although basing trust on agreement may initially seem inherently flawed—a circular form of reasoning that can only ever lead to strengthening one’s beliefs regardless of their accuracy—there are good reasons why agreement can be used as a proxy for accuracy when feedback is unavailable. Based on the principle of the “wisdom of crowds” [19], categorical decisions driven by a shared truth and unshared error will agree more frequently the greater the accuracy of the decisions (because they depend more upon the shared truth which brings them together than the unshared errors which may drive them apart) [1]. In the absence of shared bias, agreement can therefore act as an indicator of accuracy: for any better-than-chance decision-maker of fixed ability, the agreement rate of an advisor will be proportional to the accuracy of the advisor. This relationship potentially enables learning about the quality of advice even without any objective indicators. Consistent with this idea, experimental participants have been shown to distinguish good versus bad advisors even when feedback is absent [5, 20], and can even show sensitivity to features as subtle as the calibration between an advisor’s confidence and their objective accuracy [18]. However, the validity of agreement as an indicator of advice accuracy depends on the advisor and advisee making independent judgements: [1] If they share biases, or if the advisor deliberately provides advice that is tailored to the advisee’s pre-existing beliefs, then people will tend to over-trust advisors relative to their objective accuracy. Such distortions of trust have been observed experimentally [18].

Here, we investigate whether corresponding effects extend into the domain of advisor choice. Will people prefer to receive advice that is more likely to agree with them than advice that may be less palatable but objectively more useful? In agent-based modelling of network dynamics, such biases in source selection have been shown to accelerate and accentuate the tendency of individuals to form self-reinforcing echo chambers of beliefs [18, 21], indicating the potentially significant impact of these biases. However, empirical evidence for this kind of source selection behaviour, for example in studies of real-world media consumption, has been somewhat mixed. The evidence that people do tend to seek out information from sources likely to agree with them is moderate (‘selective exposure’ [22]). The evidence that people avoid information likely to disagree is poor (‘selective avoidance’ [23, 24]) with evidence becoming less persuasive as tasks become more ecologically valid [25, 26].

A priori, cases can be made for biases both towards and against selecting sources that tend to agree with one’s pre-existing beliefs. It would make sense that people seek out information they are more likely to use, because all information acquisition comes with some kind of cost, even if only attentional and opportunity costs, and rational actors should maximise their benefit-cost trade-off. It may make sense for people to seek out information they are likely to agree with, regardless of usefulness, because they may be exercising critical vigilance over their own side in a debate or serving non-informational needs such as interacting with a community who share their opinions (i.e., homophily [27]). It would also make sense, however, for people to seek out information from sources they disagree with: perhaps those we disagree with have access to evidence or reasons we had not considered; or perhaps learning about others’ views will allow us to better counter them and convert their adherents [28]. People may even prefer a balanced or random diet of information because they feel unable to judge relative quality, or because all the reasons above are pulling them in different directions.

We report a series of nine experiments in which participants learned about a pair of advisors and were then given the opportunity to choose between them. The advisors were computerised agents whose advice was programmed to vary in accuracy and/or to predominantly either agree or disagree with the participants’ choice. We hypothesised that participants would prefer to receive advice from advisors who agreed with them more often, and that this effect would be reduced or even absent when feedback was provided during the learning phase.

The experiments used two different tasks. To make contact with our previous work [18], some experiments used a simple perceptual decision task in which participants were asked on each trial to judge which of two on-screen boxes contained more dots, both before and after receiving advice from a virtual advisor. This “Dots Task” was performed without feedback, extending our previous work to ask whether, even without an objective standard against which to judge advice, participants would develop preferences for accurate and agreeing advice as seen in studies of trust and advice utilisation. A limitation of this task is its artificial (simple and highly repetitive) nature. We therefore ran a corresponding series of experiments with a general knowledge task that asked participants about the dates of historical events. We were interested in testing whether corresponding advisor preferences would be robustly observed in this “Dates Task” which involved many fewer trials but more meaningful content. Across experiments, we also varied whether the Dates Task was performed with or without objective feedback, enabling us to assess the impact of feedback on preferences for agreeing and accurate advice.

## Materials and methods

### Ethics

Ethical approval for the studies reported here was granted by the University of Oxford Medical Sciences Interdivisional Research Ethics Committee (References: R55382/RE001; R55382/RE002).

Data were collected on the Prolific web platform in phases (typically lasting a few hours per experiment), between 16th of September 2018 and 23rd June 2020.

Participants provided consent by checking checkboxes on an online form. All participants were at least 18 years of age, confirmed by the requirements for possessing an account on the Prolific platform and by explicit confirmation when giving informed consent.

### Participants

Before exclusions, we collected data over the internet from 416 participants across these nine experiments. We deliberately did not collect demographic data from participants so we do not detail their ages, gender identity, location, eyesight, or language ability. Participants were excluded from the Dots Task for: having initial accuracy below 60% or above 85% (N = 1); having a confidence distribution that was too compressed or too skewed to generate contingent advice (N = 52). Participants were excluded from the Dates Task for: having too many trials with data saving issues, or that took too long (N = 0); adjusting their after advice on fewer than 10% of trials (N = 1); using translation software to translate questions (as evident in changes to the HTML of the response button clicked; N = 3). Overall, 56 participants were excluded (some participants met multiple exclusion criteria). Data from 360 participants formed the data set analysed in the results section below.

Participants were recruited to the experiment via the Prolific Academic participant recruitment platform (https://prolific.co). Participants were prevented from taking the experiment if they had participated in one of the other experiments in the series, or if they had an overall approval rating on Prolific of less than 95/100. After reading the description of the experiment and accepting the work on the recruitment platform, participants took part in the experiment by clicking a link to the website that housed the experiment, hosted on our lab web server. Upon landing on the experiment page, participants were directed to the participant information and consent pages. After consent was obtained, they were routed to the correct experiment page, and the experiment began. After completing the experiment, participants were provided with an alphanumeric code they could submit on Prolific in exchange for payment. Participants were paid approximately £15/hour. The Dots Task experiments lasted approximately 39 minutes (minimum = 12; median = 36; maximum = 88) and the Dates Task experiments lasted approximately 17 minutes (minimum = 7; median = 15; maximum = 55). Dates Task experiments included attention checks which terminated the study as a consequence for failure; participants excluded in this way are not detailed in the exclusions above.

#### Sample size

Participants were recruited to Dots Task experiments in batches until the pre-specified number (50 participants) passed exclusion checks. The number was chosen to provide a reasonably precise characterisation of people’s preferences on the task, and give a high power for detecting a small effect (around *d* = 0.1). In this manuscript, because the results are qualitatively identical, participants over-recruited for this process are included. Participants were recruited to the Dates Task according to a Sequential Bayes Factor approach [29] that symmetrically evaluates the relative likelihood of the core hypothesis for the study and the null hypothesis. Specifically, data collection ceased when the Bayes factor for a *t*-test of advisor choice rates versus chance was greater than 3 or less than 1/3 for both feedback and no feedback conditions. The Bayesian stopping rule was introduced for the Dates Task data collection to avoid unnecessary expenditure.

### Procedure

Nine experiments were conducted, all with the same overall structure comprising two key phases. In the familiarisation phase participants performed the task while receiving advice from two advisors in separate blocks (order counterbalanced across participants), which gave them the opportunity to learn about the properties and quality of the advice on offer. Then, in the test phase, they performed a series of trials in which they were able to choose which of their two advisors to receive advice from on each trial. The nine experiments were created via factorial combination of the task performed (A. Dots Task, B. Dates Task without feedback, or C. Dates Task with feedback) and the pairing of advisors experienced (1. low versus high accuracy; 2. low versus high agreement; 3. high accuracy versus high agreement—details below). Thus, Experiments 1A, 2A and 3A used the Dots Task, Experiments 1B, 2B and 3B used the Dates Task without feedback, and Experiments 1C, 2C and 3C used the Dates task with feedback, with the respective experiments (1–3) in each series differing according to the types of advisors experienced. The Dots Task was not performed with feedback, because our lab has previously reported experiments contrasting the presence and absence of feedback using this task [18].

**Fig 1:**
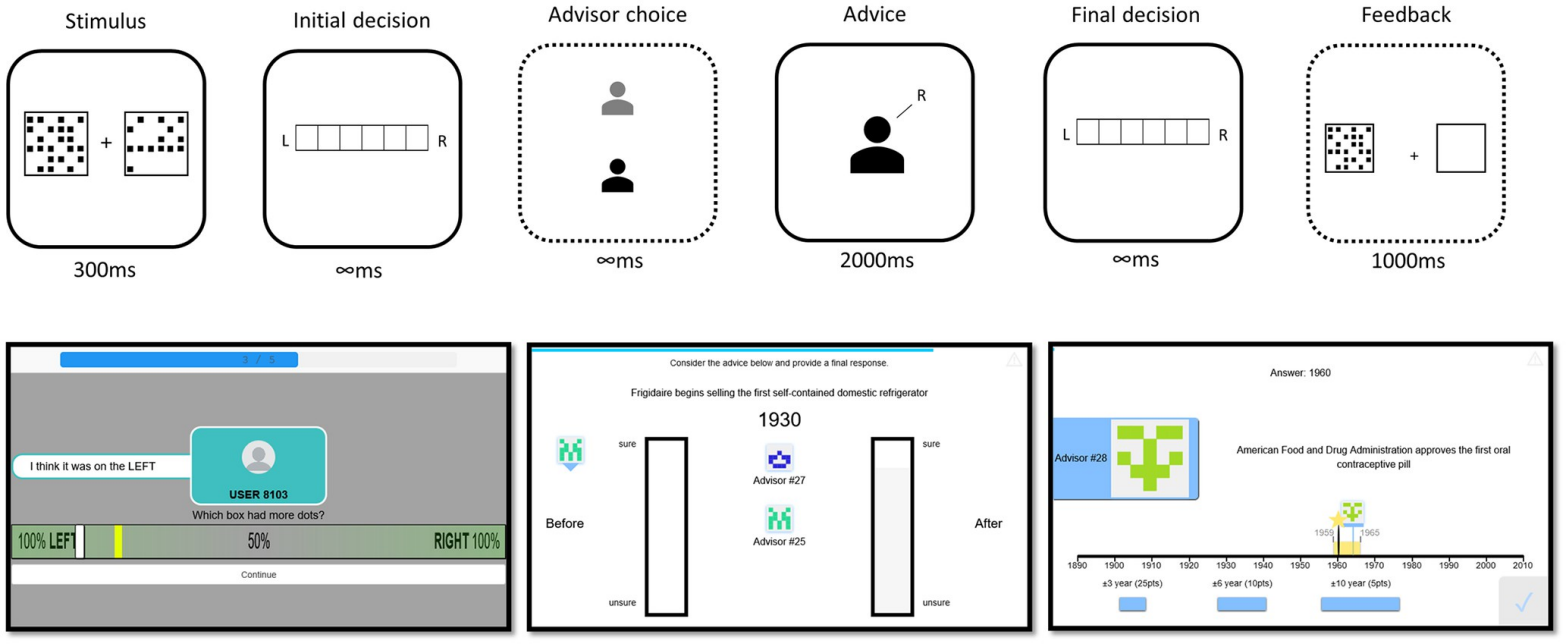
Task structures. Top: Trial sequence for the Dots Task. The trial sequence for the other tasks was the same, except that the stimulus remained visible throughout the trial. The phases with dotted outlines were not present on all trials: Advisor was pre-determined in familiarisation blocks and advisor choice was only given in test trials; feedback was only provided in Experiments 1C, 2C and 3C. Bottom: Screenshots of the different tasks. Left—Dots Task final decision screen (advice remains visible, alongside the yellow previous decision marker and the white final decision marker). Middle—Dates Task final decision screen showing the trial’s question and participants’ response options (“Before” and “After”), the participant’s initial answer (faint shading in the “After” column), and the advisor identity and their advice (avatar on the left indicates advised answer of “Before”). Each of these elements appeared sequentially as the trial progressed. Right—Continuous Dates Task feedback screen showing the participant’s final decision (yellow rectangle), advisor’s advice (small advisor marker on the timeline), and feedback marker (yellow star, answer written at the top of the screen), with these elements appearing sequentially as the trial progressed.

Trials of the Dots Task and the Dates Task both followed the same general Judge-Advisor System structure [5]. On each trial, participants made an initial decision on the decision-making task. Next, participants received advice, after which they made a final decision. Participants indicated their decisions by entering a combined answer and confidence judgement. On final decisions, participants could see their initial estimate marked on the response scale. In experiments using the Dates task with feedback, participants were then told whether this final decision was correct or incorrect. In other experiments, the next trial followed immediately after participants final decision. An overview of the tasks is shown in Fig 1.

In familiarisation blocks, participants received advice from one of two fixed advisors, whereas in test blocks the participants could choose which of the two advisors to receive advice from on each trial after making their initial estimate. After seeing the advice, participants input a final decision. The advice was always computer-generated, with the specifics of the generating procedure varied between experiments to produce advisors with different styles of advice-giving.

After all trials were completed, debrief questions were presented and feedback provided concerning the participant’s performance, including a stable link to the feedback and a payment code. The debrief questions included a questionnaire asking participants to rate their advisors’ likeability, ability, and benevolence [30], and offering them the opportunity to provide free-text comments on the advisors and the experiment in general. These questionnaire responses are not analysed because they were generally uninformative.

#### Dots Task

The Dots Task (Experiments 1A, 2A, and 3A) allows for precise control over the objective accuracy of a participant’s answers, while allowing for a fairly broad range of subjective confidence. This is the task used in the work we were attempting to extend [18].

The decision-making task in the Dots Task was judging which of two briefly (300ms) and simultaneously presented boxes contained more dots [6, 31–33]. The dots did not move during the presentation of the stimulus. The Dots Task includes a staircasing procedure that varies the difference in the number of dots in each box dynamically using a 2-up 1-down procedure. The staircasing procedure results in participants converging on an initial estimate accuracy of around 71%. The number of dots was determined by the difficulty of the trial *d*: the box with the least dots had 200—*d* dots, while the box with the most had 200 + *d* dots.

The Dots Task began with a simple interactive tutorial explaining the task and the response bar, followed by two practice blocks of 60 trials so that participants could get used to the task and the staircasing algorithm could titrate the difficulty appropriately (the staircasing continued during the remainder of the experiment). Participants then performed 5 trials with advice to get used to receiving and using advice (from different advisors to those appearing in the main experiment). The main experiment consisted of three blocks: in the first two of these participants were advised by one advisor; and in the final block they were able to choose between their two advisors on each trial. Each experimental block contained 60 trials. The exception to this rule is that the experiment contrasting high accuracy and high agreement advisors had only 4 practice advice trials and 30 choice trials. We shortened the duration of Experiments 3B and 3C having noted in pilot versions of the continuous dates task that participants’ advisor preferences were apparent in their choices within the first 30 trials.

#### Dates Task

The Dates Task (Experiments 1B, 1C, 2B, and 2C) is shorter and more engaging than the Dots Task, as well as being more similar to tasks used in previous Judge-Advisor System implementations [5, 34].

The Dates Task used a decision-making task that required participants to identify whether 20th Century events occurred before or after a particular date. In each trial, participants saw a text representation of an event that occurred in a specific year of the 20th Century, for example “Roger Bannister runs the first 4-minute mile”, which occurred in 1954; or “US-led coalition expels the Iraqi Army from Kuwait in Operation Desert Storm”, which occurred in 1991. The participant also saw a date written below the event, and two bars labelled “Before” (on the left) and “After” (on the right). The bars were vertically oriented to emphasise that participants’ answers should indicate their binary before/after decision and associated confidence, whereas a horizontal confidence scale (as used in the Dots task) might be mis-interpreted as a timeline centred around the comparison date. Participants indicated their answers by selecting one of the bars, choosing a point higher up the bar the more confident they were in their choice. Advice comprised the advisor’s avatar presented on either the left (advising “Before”) or right (advising “After”) of the screen as show in Fig 1.

The (categorical) Dates Task started with an interactive tutorial, 10 warm-up questions to give participants a chance to calibrate their confidence, and a tutorial on advice. Thereafter the participants performed three blocks of experimental trials. In the first two blocks, participants were advised by a single advisor (order counterbalanced across participants), and in the final block they were able to choose between those advisors. The familiarisation blocks where advisors were assigned automatically consisted of 15 trials, and the test block containing a choice of advisors contained 10 trials. During warm-up trials and, for some groups of participants also during familiarisation block trials, feedback was provided following participants’ final decisions. Feedback was presented as a gold star displayed on the correct side of the screen, with the actual year the event occurred printed below.

#### Continuous dates task

Our final experiments (3B and 3C) used a variant of the Dates Task involving estimation on a continuous scale, to provide an open-ended question to participants that is more closely modelled on previous Judge-Advisor System work [5, 34, 35]. This version of the task allowed to us to create high agreement and high accuracy advisors that presented clearly distinct advice profiles to participants, even though the task had relatively few trials and gave us relatively little control over participant accuracy (which varied depending on participants’ idiosyncratic historical knowledge).

Unlike the other tasks, the Dates Task version of the high accuracy versus high agreement used a continuous response rather than a binary decision with confidence. Participants saw questions from the same set as in the categorical version of the task, but did not see a date. Instead, participants indicated their decision by dragging a marker onto a timeline so that it covered a span of years. Participants could choose from markers that were 7, 13, and 21 years wide, worth 25, 10, and 5 points, respectively. Points were scored on trials where the participant’s marker covered the year in which the event actually occurred. Points were arbitrary, and unrelated to remuneration, but were included to encourage participants to vary marker size as a function of their confidence in their answer. Advice in this task comprised a timeline marker identified by the advisor’s avatar image. Feedback, if provided, consisted of text at the top of the screen indicating the correct answer year and a gold star placed on the timeline in the location corresponding with the answer.

The experiment trial structure was identical to that for the categorical Dates Task, with the exception that some of the participants experienced only 10 trials in each familiarisation block because the preregistered replication (see below) for this experiment was shortened.

### Advice

The advice was computer-generated throughout all the experiments. The advisors gave advice that depended upon the objective answer and/or the participant’s initial estimate. Advice was conceptualised along two dimensions: accuracy and agreement. For the Dots Task and the categorical version of the Dates Task, advice was considered ‘accurate’ if the advice endorsed the option that was objectively correct. Conversely, advice was considered ‘agreeing’ if the advice endorsed the same option the participant chose as their initial estimate, regardless of the accuracy of that initial estimate.

For advisors with fixed accuracy profiles, agreement with the participant was probabilistic and contingent on the correctness of the participant’s initial estimate, producing advice profiles detailed in Table 1. Reflecting straightforward probabilities, when participants performed the task at above-chance levels in their initial decisions, high accuracy advisors were more likely to agree with those initial decisions than were low accuracy advisors. By hypothesis, it is these differences in agreement rate that allow participants to distinguish high vs low accuracy advisors even in the absence of objective feedback.

**Table 1:** Advice calculations.

		Agreement when participant is		Overall	
Expt.	Advisor	Correct (%)	Incorrect (%)	Accuracy (%)	Agreement (%)
1A	High Accuracy (Dots)	80	20	80.00	62.60
1A	Low Accuracy (Dots)	60	40	60.00	54.20
2A	High Agreement (Dots)	84	61	70.95	77.33
2A	Low Agreement (Dots)	66	17	70.93	51.79
3A	High Accuracy* (Dots)	80	20	80.00	62.60
3A	High Agreement* (Dots)	80	80	62.60	80.00
1B, 1C	High Accuracy (Dates)	80	20	80.00	55.40
1B, 1C	Low Accuracy (Dates)	59	41	59.00	51.62
2B, 2C	High Agreement (Dates)	90	65	67.45	79.75
2B, 2C	Low Agreement (Dates)	75	35	70.90	58.60
**Expt.**	**Advisor**			**Accuracy (years)**	**Agreement (years)**
3B, 3C	High Accuracy (Dates (cont.))	-	-	3.99	15.76
3B, 3C	High Agreement (Dates (cont.))	-	-	15.76	3.99

The values for overall agreement and accuracy percentages correspond to programmed targets and their derived consequences based on the participants’ responses. The “years” values for Experiment 3B, 3C advisors correspond to the average deviation from both the correct answer (Accuracy) and answers that participants provided in the experiment (Agreement).

The accuracy of participants in the Dots Task was constrained to 71% by a staircasing procedure. The accuracy of participants in the Dates Task was not controlled, but was observed to be 59% overall.

* These advisors were used in the experiment contrasting high accuracy versus high agreement advisors.

For advisors with fixed agreement profiles, the objective accuracy of their advice depended on the accuracy of participants’ initial decisions. Because Dots Task accuracy was carefully controlled via an adaptive staircase, we could design agreement rates so that the objective accuracy of advice was well matched for high versus low agreement advisors. Participants’ accuracy in the Dates Task was less well controlled because it depended on their particular historical knowledge and their luck when guessing, and so accuracy was less well matched for high versus low agreement advisors in this task.

In the continuous version of the Dates Task (which featured high accuracy versus high agreement advisors), the advisors’ accuracy and agreement were taken as continuous properties, measured from the centre of the advisor’s marker to the correct year (for accuracy) or the centre of the participant’s initial estimate marker (agreement). The high accuracy advisor’s advice was drawn from a normal distribution around the correct year, while the high agreement advisor’s advice was drawn from a normal distribution around the participant’s initial estimate (standard deviation = 5 years). On 13.5% of trials, advisors gave advice that was neither correct nor agreeing, by drawing from a normal distribution around a point on the opposite side of the correct answer from the initial estimate (so that, for example, if a participant estimated the date of Roger Bannister’s historic run as 1960, the centre of the advice distribution would fall at 1948, symmetrically opposite the estimate around the reference point of 1954). These trials were included to provide a subset of trials on which advisor influence might be measured without the confound of the advice itself (which differed across the two advisors by design). Analyses of advisor influence are not reported here (but can be found in [36]).

### Preregistration

Each individual experiment was preregistered using the Open Science Framework (https://osf.io/), with the exception of the Dots Task experiment with high agreement versus low agreement advisors (Experiment 2A), which was not preregistered due to an oversight. The preregistration links are available in Table 2. One additional experiment not reported here, testing a more complex insight derived from [18], is reported in S3 File.

**Table 2:** Preregistration links for each experiment.

Advisors	Dots Task	Dates Task
High accuracy versus low accuracy	1A	1B, 1C
	https://osf.io/u5hgj	https://osf.io/5xpvq
High agreement versus low agreement	2A	2B, 2C
	*Omitted by oversight*	https://osf.io/8d7vg
High accuracy versus high agreement	3A	3B, 3C
	https://osf.io/f3k4x	https://osf.io/nwmx5

#### Deviations

There were several deviations from the preregistered plan for these experiments. Firstly, preregistrations for the Dots Task stated that participants beyond the stated sample size of 50 would be dropped. In this paper we include the participants who would have been excluded on the basis of this criterion. Excluding participants according to the preregistered plan produced the same results. Similarly, the Dates Task in which participants chose between high agreement and high accuracy advisors was conducted as two separate studies: a pilot experiment and a preregistered replication. In this paper we combine the data for these two experiments to streamline the reporting process (the results in the experiments when analysed individually are highly similar and qualitatively identical).

The statistical analyses presented in this paper are frequentist one-sample *t*-tests (see below for details). For the Dates Task experiments, Bayesian *t*-tests were used to assess whether or not pick rates differed from chance, and data ceased to be collected when sufficient evidence was accumulated to support either the presence or absence of such a difference. Frequentist tests are presented here for clarity and consistency, and we note that the results of the frequentist test and the Bayesian equivalents are qualitatively identical.

## Open materials

The materials used for running these studies are available for reuse under an MIT license. The software is housed in a GitHub repository (https://github.com/oxacclab/ExploringSocialMetacognition). Different experiments took place at different times, while the software was changing. Links to the repository state as it was at the time each experiment was run are included in the supporting information (S1 File).

## Statistical analysis

The results of each study are presented as two-sided one-sample Student’s *t*-tests, with alpha set to 0.05. No correction is made for multiple comparisons because each test pertains to a specific hypothesis within a discrete experiment. All tests are reported with the structure *t*(DF) = X, *p* = X, *μ* = X[X, X] vs *μ*_0_ = X, *d* = X where X stands for the number in question, DF gives the degrees of freedom of the *t*-test, *p* the *p*-value, *μ* the sample mean with 95% confidence intervals in square brackets, *μ*_0_ the value to which the sample mean is compared, and *d* the Cohen’s *d* measure of effect size. Conditional means are reported as *M*_*condition*_ or *M*_*conditionA*|*conditionB*_ for interactions.

Analysis was conducted in R version 4.1.0 [37], and the manuscript was prepared in Rstudio (version 1.4.1717) [38] using the knitr [39] and tidyverse [40] packages. A complete list of packages and versions used, and other software environment details, are included in the supporting information (S2 File). The analyses can be run using the code provided in the source code repository for this manuscript (https://github.com/oxacclab/advisor-choice-paper).

## Results

Participants completed on-line tasks in which they were familiarised with two advisors and then allowed to choose between them. We examined three advisor contrasts: an accurate versus an inaccurate advisor; a low versus high agreement advisor; and an accurate versus an agreeing advisor. We hypothesised that participants would prefer to receive advice from the advisors who agreed with them more often, and that this effect would reduce or even disappear when feedback was provided during the learning phase. Before presenting analyses relating to these key predictions about advisor choice, we first present analyses of basic task performance across experiments, to establish the success of advisor accuracy and agreement manipulations and to establish that participants paid attention to the advice offered.

### Task accuracy and advice usage

Our initial analyses focused on task performance in the familiarisation phase, as participants had the opportunity to make decisions with advice (and, in some experiments, with trialwise feedback). Participants averaged 71% correct initial decisions in the Dots Task (Experiments 1A, 2A and 3A), the target value of the adaptive staircase procedure used, and averaged 59% correct decisions in the categorical Dates Task (Experiments 1B-C, 2B-C). Participants in the continuous version of the Dates Task (Experiments 3B-C) initially placed their chosen marker such that it included the actual year of the event described on 42% of trials.

Participants gave meaningful confidence reports. As a representative example for the Dots Task, in Experiment 1A, participants reported higher confidence in perceptual decisions that were objectively correct than incorrect, *F*(1, 49) = 168.89, *p* < .001; *M*_*Correct*_ = 23.64, *M*_*Incorrect*_ = 16.40, an effect that was more marked for final (post-advice) decisions than initial (pre-advice) ones, as shown in the difference in confidence (when Correct—when Incorrect) *F*(1, 49) = 46.01, *p* < .001; *M*_*Difference*|*Final*_ = 10.34, *M*_*Difference*|*Initial*_ = 4.14. Similar effects were observed in the categorical version of the Dates Task. In Experiments 1B-C, for example, participants reported higher confidence in before/after judgments that were correct versus incorrect, *F*(1, 61) = 102.69, *p* < .001; *M*_*Correct*_ = 55.84, *M*_*Incorrect*_ = 36.41, with larger differences seen post-advice, *F*(1, 61) = 68.17, *p* < .001; *M*_*Difference*|*FinalDecision*_ = 34.56, *M*_*Difference*|*InitialEstimate*_ = 4.31. In the continuous version of the Dates Task (Experiments 3B-C), mean error of initial estimates varied with the width of the marker (equivalent to a subjective confidence interval) chosen by participants: *M*_*Error*|*Marker*7_ = 11; *M*_*Error*|*Marker*13_ = 16; *M*_*Error*|*Marker*21_ = 19.

The changes in confidence from pre- to post-advice give an indication that participants generally paid attention to advice and made use of it. More direct evidence comes from the observation that participants’ decisions were more accurate after than before advice. This improvement was clear in Experiments 1A-C, in which advice was tethered to the objectively correct answer (1A: *F*(1, 49) = 4.80, *p* = .033; *M*_*Final*_ = 0.72, *M*_*Initial*_ = 0.71; 1B-C: *F*(1, 61) = 36.40, *p* < .001; *M*_*FinalDecision*_ = 0.68, *M*_*InitialEstimate*_ = 0.60), and more so after high accuracy than low accuracy advice (1A: *F*(1, 49) = 17.32, *p* < .001; *M*_*Improvement*|*HighAccuracy*_ = 0.03, *M*_*Improvement*|*LowAccuracy*_ = −0.01; 1B-C: *F*(1, 61) = 32.46, *p* < .001; *M*_*Improvement*|*HighAccuracy*_ = 0.15, *M*_*Improvement*|*LowAccuracy*_ = 0.01). Interestingly, participants also benefited from advice even when it was tethered (via agreement rates) to their own initial decisions. This effect was significant overall in Experiment 2A, *F*(1, 49) = 5.48, *p* = .023; *M*_*Final*_ = 0.73, *M*_*Initial*_ = 0.72, where it did not differ consistently according to whether advisor agreement rate was low or high, *F*(1, 49) = 1.33, *p* = .255; *M*_*Improvement*|*HighAgreement*_ = 0.00, *M*_*Improvement*|*LowAgreement*_ = 0.02. Participants in Experiments 2B-C similarly used advice to improve their decisions, *F*(1, 73) = 32.19, *p* < .001; *M*_*FinalDecision*_ = 0.66, *M*_*InitialEstimate*_ = 0.60, and here the post-advice increase in decision accuracy was greater when paired with the Low agreement advisor than the High agreement advisor, *F*(1, 73) = 5.30, *p* = .024; *M*_*Improvement*|*HighAgreement*_ = 0.04, *M*_*Improvement*|*LowAgreement*_ = 0.09, reflecting the slightly greater objective accuracy of this advice, particularly on trials when the participant’s own initial decision was wrong (see Table 1). In Experiments 3A-C, the overall benefit of advice (3A: *F*(1, 63) = 23.20, *p* < .001; *M*_*Final*_ = 0.74, *M*_*Initial*_ = 0.72; 3B-C: *F*(1, 32) = 69.02, *p* < .001; *M*_*Initial*_ = 15.84, *M*_*Final*_ = 10.28) was much greater when this advice came from a high accuracy advisor than a high agreement advisor (3A: *F*(1, 63) = 50.28, *p* < .001; *M*_*Improvement*|*HighAccuracy*_ = 0.05, *M*_*Improvement*|*HighAgreement*_ = −0.01; 3B-C: *F*(1, 32) = 60.26, *p* < .001; *M*_*Reduction*|*HighAccuracy*_ = 9.67, *M*_*Reduction*|*HighAgreement*_ = 1.46).

Thus, in the familiarisation phase of the experiment, participants experienced different advice profiles, tried to use advice to improve their decisions, and benefited more from advice that was tethered to the objectively correct response than advice tethered to their own initial decisions. Of critical interest, then, was participants’ choices in test blocks of which advisors to receive advice from.

### Advisor choice

**Fig 2:**
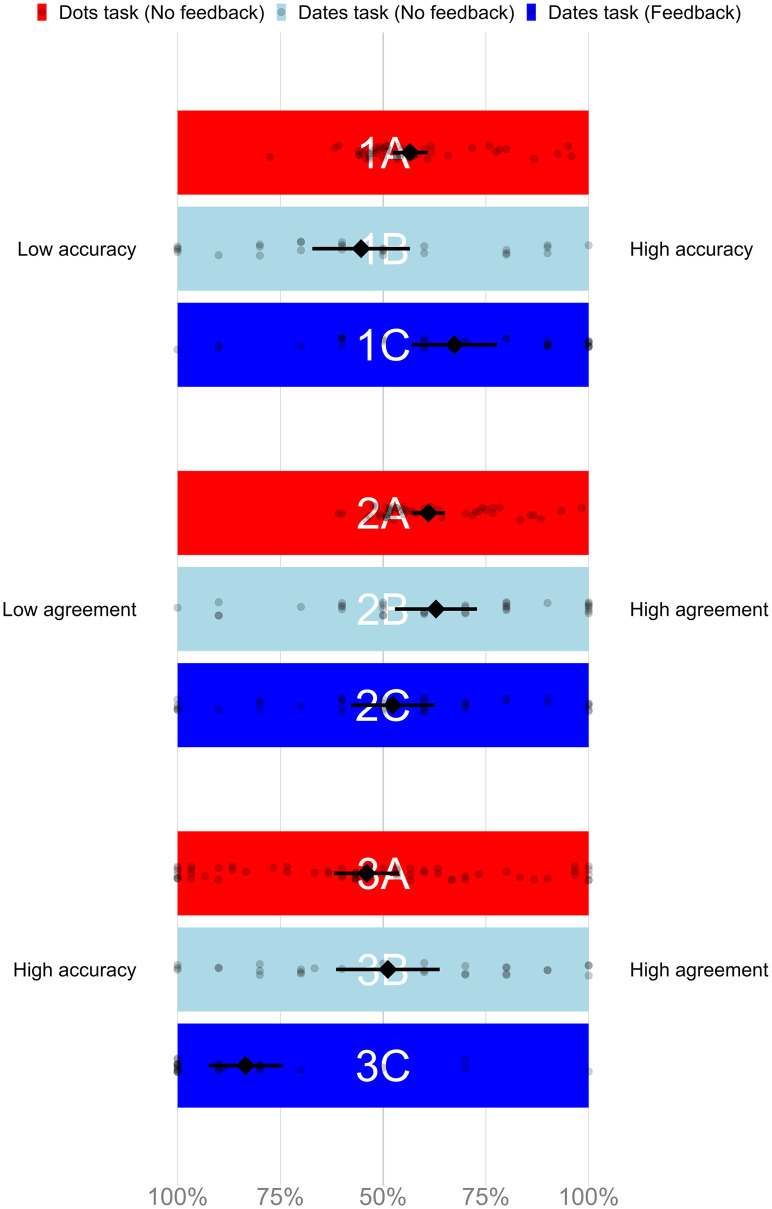
Advisor choice frequency across all tasks. Experiments were conducted using two tasks, a Dots task (blue bars) and a Dates task (red bars). In the Dates task, some participants received feedback during the familiarisation phase (light shading). Light grey points indicate the relative pick rate of the advisors for a single participant. The larger diamonds represent the mean of these individual pick rates, the 95% confidence intervals of which are represented by the error bars. Participants in the Dots Task performed 60 choice trials, while those in Dates Tasks performed 10, so Dots Task choice ratios are more granular.

Participants’ choices of advisor across the nine experiments are summarised in Fig 2. Participants frequently had strong individual preferences between advisors. We were interested in whether the relative frequency of choosing advisors differed systematically according to the type of advice the advisor gave, the task performed, and the availability of feedback. For each experiment, we tested whether observed pick rates differed from equivalent rates (50%). As shown in Fig 2, we observed a mixture of clearly different and approximately equivalent pick rates.

In the first series of experiments, participants chose between advisors who differed in objective accuracy. In the Dots Task (Experiment 1A), which did not include feedback, we observed that participants had a systematic preference for receiving advice from advisors who were more as opposed to less accurate (*t*(49) = 3.09, *p* = 0.003, *μ* = 0.57 [0.52, 0.61] vs *μ*_0_ = 0.500, *d* = 0.44). There was wide variability of the strength of this preference, with most participants showing only a slight bias towards the more accurate advisor, but with a minority showing a strong preference. These findings conceptually replicate and our earlier findings on advice utilisation [18], showing again that people are able to learn the quality of advice even in the absence of an objective standard, and extend these results to show that this learning also guides their choice of advice source. However, contrary to expectation, a corresponding preference was not apparent in the Dates Task when feedback was absent during learning (Experiment 1B, *t*(27) = −0.93, *p* = 0.363, *μ* = 0.45 [0.33, 0.57] vs *μ*_0_ = 0.500, *d* = −0.18). Here, preferences were quite evenly distributed across the full range of directions and strengths, with a slight numerical advantage for the Low accuracy advisor. The lack of consistent preference potentially indicates that participants found it difficult to distinguish advisors given their own relatively low accuracies and given relatively few familiarisation trials (15 per advisor, versus 60 in the Dots task). In contrast, the Dates Task with feedback (Experiment 1C) showed a more consistent pattern, with participants systematically preferring to receive advice from a more rather than less accurate advisor (*t*(33) = 3.41, *p* = 0.002, *μ* = 0.67 [0.57, 0.78] vs *μ*_0_ = 0.500, *d* = 0.58). The between-experiment comparison of Experiments 1B and 1C was significant (*t*(57.02) = −2.95, *p* = 0.005, *M*_*feedback*_ = 0.67 [0.57, 0.78], *M*_*nofeedback*_ = 0.45 [0.33, 0.57], |*d*| = 0.71).

The second series of experiments focused on the effect of advisor agreement. In the Dots task (Experiment 2A), participants had a systematic preference for receiving advice from advisors who were more as opposed to less agreeing (*t*(49) = 5.43, *p* < 0.001, *μ* = 0.61 [0.57, 0.65] vs *μ*_0_ = 0.500, *d* = 0.77). As with the accuracy preference in this task, the pattern across participants was of a majority showing only a small preference and a sizeable minority showing stronger preferences. In the Dates Task, when feedback was not provided during learning (Experiment 2B), we saw systematic preferences emerge that favoured the agreeing advisor (*t*(34) = 2.62, *p* = 0.013, *μ* = 0.63 [0.53, 0.73] vs *μ*_0_ = 0.500, *d* = 0.44), as expected. In contrast to the Dots Task version, some Dates Task participants chose the low agreement advisor on the majority of test trials, but the average preference was numerically larger in the Dates Task overall. In the Dates Task with feedback (Experiment 2C), participants did not appear to have a systematic preference for advice that agreed more rather than less (*t*(38) = 0.46, *p* = 0.648, *μ* = 0.52 [0.42, 0.62] vs *μ*_0_ = 0.500, *d* = 0.07), with the full range of preferences—ranging from always selecting the low agreement advisor to always selecting the high agreement advisor—seen across participants. Collectively, these findings are consistent with our key predictions that participants will exhibit a preference for advisors who have previously tended to agree with a participant’s own initial decisions, but that this tendency will be reduced or even absent if feedback is provided. However, the between-experiment comparison of Experiments 2B and 2C was not significant (*t*(71.91) = 1.5, *p* = 0.137, *M*_*feedback*_ = 0.52 [0.42, 0.62], *M*_*nofeedback*_ = 0.63 [0.53, 0.73], |*d*| = 0.35), reflecting the high degree of inter-individual variability in preferences apparent in both experiments.

Our underlying theory suggests that participants learn to trust accurate advisors over less accurate advisors when feedback is absent because the accurate advisors agree with them more frequently. If this were the case, we might also expect an agreeing advisor to be preferred to an accurate advisor when directly contrasted. However, we did not observe this expected effect in our final series of experiments, either with the Dots Task (Experiment 3A: *t*(63) = −1.01, *p* = 0.317, *μ* = 0.46 [0.38, 0.54] vs *μ*_0_ = 0.500, *d* = −0.13) or the Dates Task (Experiment 3B: *t*(28) = 0.19, *p* = 0.853, *μ* = 0.51 [0.39, 0.64] vs *μ*_0_ = 0.500, *d* = 0.03). The full range of idiosyncratic advisor preferences was apparent in both datasets, with some participants choosing the high accuracy advisor on all trials, some choosing the high agreement advisor on all trials, and gradations in between—averaging to no consistent preference overall. In contrast, when feedback was provided in the Dates Task (Experiment 3C), a strong preference for the high accuracy advisor was apparent (*t*(30) = −7.63, *p* < 0.001, *μ* = 0.17 [0.08, 0.25] vs *μ*_0_ = 0.500, *d* = −1.37), the largest systematic preference across all the experiments. The between-experiment comparison of Experiments 3B and 3C was reliable (*t*(51.31) = 4.58, *p* < 0.001, *M*_*feedback*_ = 0.17 [0.08, 0.25], *M*_*nofeedback*_ = 0.51 [0.39, 0.64], |*d*| = 1.03).

### Agreement rate as a predictor of choice

We hypothesised that participants used agreement as a proxy for assessing accuracy when accuracy information could not be derived from objective feedback. We found equivocal evidence for this when looking for systematic preferences for choosing an advisor. A more lenient test of our hypothesis is to explore whether observed agreement rate and advisor pick preference are related. We found no statistically significant correlation between experienced agreement rate and preference strength in the high versus low agreement experiments, either in the Dots Task (*r* = −0.2 [-0.45, 0.08], *p* = 0.16) or in the Dates Task without feedback (*r* = 0.18 [-0.16, 0.48], *p* = 0.301).

This should not be taken as a strong indicator that these features are unrelated, however, because the variation in experienced agreement rates was deliberately minimised, and there were no significant correlations between experienced *accuracy* and pick preference in the high versus low accuracy advisor experiments (Dots Task: *r* = 0.12 [-0.16, 0.39], *p* = 0.402; Dates Task without feedback: *r* = 0.33 [-0.05, 0.63], *p* = 0.086).

## Discussion

Participants performed online experiments in a Judge-Advisor System in which they learned about, and then chose between, advisors who varied in their tendency to agree with the participants’ initial estimates or to provide correct advice. We had expected that participants would prefer to receive advice from advisors who were more likely to agree with them when they lacked objective feedback against which to evaluate the quality of advice. Consistent with this, we observed systematic preferences for accuracy and agreement in the Dots Task, and for agreement alone in the Dates Task. There were no clear preferences when accuracy and agreement were pitted against one another. When feedback was provided, participants overwhelmingly preferred accuracy and did not seem to develop a preference for agreement. Thus, we found no consistent preference for high agreement vs low agreement advisors when feedback was provided in the Dates task (Experiment 2C), and we found much stronger preferences for high accuracy vs low accuracy advisors when feedback was provided (Experiment 1C) than when it was absent (Experiment 1B), despite agreement rate differences between advisors being the same across these two experiments.

Our underlying theory, that people use advice agreement as a proxy for accuracy when objective feedback is unavailable, was supported by some of the experimental results. The results of the Dots Task experiments were consistent with the idea that people’s assessments of accuracy are based on agreement: both agreement and accuracy led to advisors being preferred. In this way, our findings replicate previous observations that people are able to discern the quality of advice they receive even when feedback is absent [5, 20], and tend to over-trust advisors who agree with them more frequently [18], and extend these findings to show the impact of this trust extends to their choice of advisors.

Contrary to our expectations, however, when participants chose between an accurate and agreeing advisor, no systematic preferences were observed in the absence of feedback. It is possible that the setup of the experiments meant that the advice profiles of the two advisors were not sufficiently distinct for participants to learn to differentiate them within the time and trials available. This explanation seems unlikely because the magnitude of the differences along the dimensions of agreement and accuracy is similar to the magnitudes of differences in the other two experiments where effects were observed. Another explanation may be that agreeing advice as generated in these experiments provided almost no information (being mostly dependent on the participant’s initial estimate rather than the correct answer), and thus a natural correlation between accuracy and agreement was disrupted. In the absence of shared bias, agreement rates will be proportional to decision-makers’ accuracy, and accuracy for well-calibrated participants will be proportional to confidence. Thus, participants ought to expect higher agreement rates when they were more confident and lower agreement rates when they were less confident; a contingency that was not implemented in our design. Indeed, if the confidence-mediated agreement theory of trust updating [18] is correct, it is possible participants viewed agreement when they were very un-confident as a source of suspicion. It is also possible that the lack of any systematic effect in advisor preference is the result of averaging over two strong but opposed preferences, for accuracy and agreement, that here were set in opposition. This may be the case: an inspection of the data presented in Fig 2 shows individual participants’ preferences spread quite homogeneously throughout the range of possible values, suggesting some participants did have quite strong preferences for each of the advisors. This pattern is particularly notable in the Dots Task given that our other experiments with this task tended to show significant clustering around a balanced choices of advisors, suggesting that deviations from balanced choices are indicative of real preferences. Nevertheless, it remains possible that people develop unsystematic preferences for advisors based on arbitrary criteria when there is no task-relevant way of differentiating them.

The two tasks, although very different, produced overall similar results—the only major qualitative difference was a systematic preference for high over low accuracy in the Dots Task where none was found for the Dates Task. It is probable that this difference is a consequence of the structure of the tasks: the Dots Task provides much more exposure to the advisors during learning, and has many more choice trials over which preferences can be assessed. The Dates Task is also a much more difficult task, meaning that participants may have been much less able to detect the differences in agreement rates that we suggest underpin accuracy assessments. The agreement differences between the advisors in the accuracy experiments, as shown in Table 1, were around 8% in the Dots Task and 4% in the Dates Task.

Consistent across tasks was the finding that, when feedback is absent, people preferred to hear from advisors who agreed with them more frequently. The consequences of preferentially sampling from sources that are more likely to confirm one’s initial opinions are potentially harmful, from making elementary reasoning mistakes [41] through to creating polarised political environments wherein only like-minded people have a voice [2, 21]. Indeed, as shown in earlier work from our lab, using agreement as a proxy for accuracy leads to simulated agents forming echo-chambers [18]. This process is accelerated where agents selectively sample advice [21]. In this context, it is somewhat reassuring to observe that in our experiments participants only preferred agreement where the alternative was disagreement: when agreement was contrasted with accuracy participants expressed a wide range of preference strengths and directions. This finding that source selection behaviour is less confirmation-seeking than suspected ties in neatly with work in the selective approach and avoidance literature that indicates people show relatively little partisan discrimination in their media consumption [23, 25, 26, 42], and evidence in favour tends to come from more artificial rather than naturalistic designs [43]. Potentially, the tendency to prefer agreeing over accurate advice may be a stable trait subject to individual differences, perhaps related concepts such as need for cognition [44] or openness to experience (although recent work on information-seeking and openness does not support this suggestion [45]). Participants took part in only a single experiment in our study, so we are unable to explore the stability of preferences here.

## Conclusions

The present study has explored how people use their past experience of advice, either with or without the yardstick of objective feedback, to guide subsequent choice of advisors. With feedback, people naturally prefer advisors who are objectively more reliable. Absent feedback, people show a tendency to choose advisors who in the past have agreed with them more frequently (versus less frequently), an effect that may exacerbate self-reinforcing cycles of information seeking and belief updating. Surprisingly, however, we did not observe a consistent preference for advisors who tend to agree over advisors who tend to be correct when feedback is absent. The wide individual differences in preferences in this case present an intriguing avenue for future research.

## Supporting information

S1 File Software code links. Links to the GitHub repository as it stood at the time each of the experiments covered in the paper was conducted.
(TXT)

S2 File Computational environment details. System information and package version information for the R computing environment used to build this manuscript.
(TXT)

S3 File Confidence contingent advice. An additional experiment in this project contrasted advisors with more complex advice profiles. This is a brief report of that experiment.
(TXT)
